# Investigating the potential effects of selective histone deacetylase 6 inhibitor ACY1215 on infarct size in rats with cardiac ischemia-reperfusion injury

**DOI:** 10.1186/s40360-020-0400-0

**Published:** 2020-03-12

**Authors:** Chao-Feng Lin, Kai-Cheng Hsu, Wei-Chun HuangFu, Tony Eight Lin, Han-Li Huang, Shiow-Lin Pan

**Affiliations:** 1 Ph.D. Program for Cancer Molecular Biology and Drug Discovery, College of Medical Science and Technology, Taipei Medical University and Academia Sinica, Taipei, Taiwan; 2 Department of Medicine, MacKay Medical College, New Taipei City, 252 Taiwan; 3 Division of Cardiology, Department of Internal Medicine, MacKay Memorial Hospital, Taipei, 104 Taiwan; 4 TMU Biomedical Commercialization Center, Taipei Medical University, Taipei, 110 Taiwan; 5 Graduate Institute of Cancer Molecular Biology and Drug Discovery, College of Medical Science and Technology, Taipei Medical University, Taipei, 110 Taiwan; 6 Program in Biotechnology Research and Development, College of Pharmacy, Taipei Medical University, Taipei, 110 Taiwan

**Keywords:** Myocardial infarction, Ischemia-reperfusion injury, Histone deacetylase 6 inhibitor, Hypoxia inducible factor-1α, Infarct size

## Abstract

**Background:**

Despite the fact that histone deacetylase (HDAC) inhibitors have been tested to treat various cardiovascular diseases, the effects of selective HDAC6 inhibitor ACY1215 on infarct size during cardiac ischemia-reperfusion (IR) injury still remain unknown. In the present study we aimed to investigate the effects of ACY1215 on infarct size in rats with cardiac IR injury, as well as to examine the association between HDAC6 inhibitors and the gene expression of hypoxia inducible factor-1α (HIF-1α), a key regulator of cellular responses to hypoxia.

**Methods:**

By using computational analysis of high-throughput expression profiling dataset, the association between HDAC inhibitors (pan-HDAC inhibitors panobinostat and vorinostat, and HDAC6 inhibitor ISOX) and their effects on HIF-1α gene-expression were evaluated. The male Wistar rats treated with ligation of left coronary artery followed by reperfusion were used as a cardiac IR model. ACY1215 (50 mg/kg), pan-HDAC inhibitor MPT0E028 (25 mg/kg), and vehicle were intraperitoneally injected within 5 min before reperfusion. The infarct size in rat myocardium was determined by 2,3,5-triphenyltetrazolium chloride staining. The serum levels of transforming growth factor-β (TGF-β) and C-reactive protein (CRP) were also determined.

**Results:**

The high-throughput gene expression assay showed that treatment of ISOX was associated with a more decreased gene expression of HIF-1α than that of panobinostat and vorinostat. Compared to control rats, ACY1215-treated rats had a smaller infarct size (49.75 ± 9.36% vs. 19.22 ± 1.70%, *p* < 0.05), while MPT0E028-treated rats had a similar infarct size to control rats. ACY-1215- and MPT0E028-treated rats had a trend in decreased serum TGF-β levels, but not statistically significant. ACY1215-treated rats also had higher serum CRP levels compared to control rats (641.6 μg/mL vs. 961.37 ± 64.94 μg/mL, *p* < 0.05).

**Conclusions:**

Our research indicated that HDAC6 inhibition by ACY1215 might reduce infarct size in rats with cardiac IR injury possibly through modulating HIF-1α expression. TGF-β and CRP should be useful biomarkers to monitor the use of ACY1215 in cardiac IR injury.

## Background

Myocardial infarction (MI), mainly caused by coronary artery occlusion, is one of the most life-threatening diseases in the world [1]. Despite successful reperfusion of occluded coronary arteries, ischemic cardiomyocyte death followed by reperfusion may result in ischemia-reperfusion (IR) injury that lead to expansion of infarct size, post-MI cardiac fibrosis, and ventricular dysfunction [2, 3]. The myocardium jeopardized in IR injury is characterized by an enhanced expression of transforming growth factor-β (TGF-β), myofibrillar destruction, and infiltrating leukocytes. These mentioned histological signs become more manifest during reperfusion than that during ischemia [2, 4]. The transcriptional complex hypoxia inducible factor-1α (HIF-1α) and TGF-β have been reported to be key regulators of the cellular and metabolic alteration during MI [5, 6]. Additionally, HIF-1α and TGF-β may play synergetic roles in infarct size and cardiac fibrosis following MI [5, 6]. Therefore, pharmacological interventions to reduce infarct size by modulating the expression of HIF-1α and TGF-β are potential strategies to diminish cardiac IR injury and preserve ventricular function.

Epigenetic modification in gene expression and cellular responses by histone deacetylase (HDAC) has gained much attention in recent years and HDAC inhibitors have been tested to treat various diseases [7, 8]. Currently, 18 mammalian HDACs have been identified and grouped into 4 classes (Class I: HDAC1, HDAC2, HDAC3, and HDAC8; Class IIa: HDAC4, HDAC5, HDAC7, and HDAC9; Class IIb: HDAC6 and HDAC10; Class III: sirtuins 1–7; Class IV: HDAC11) [9]. Vorinostat, a pan-HDAC inhibitor, has been approved for the treatment of patients with cutaneous T-cell lymphoma [7]. Recently we also identified a pan-HDAC inhibitor MPT0E028 that has a more potent anticancer activity compared to vorinostat [10]. In addition to pan-HDAC inhibitors, selective HDAC6 inhibitor ACY1215 has also shown anticancer effects [11] on reducing the cell proliferation of colon cancer cells [11].

Whether HDAC inhibitors have cardioprotective effects on limiting infarct size in cardiac IR injury is recently under investigation. In our recent research we observed that MPT0E028 significantly reduced the serum N-terminal prohormone of brain natriuretic peptide (NT-proBNP) levels and collagen area in myocardium of isoproterenol (ISO)-treated rats (Supplementary Figure 1, and 2) (Data not published). Because the animal model of ISO-treated rats has been validated to show a histological presentation of MI and post-MI cardiac fibrosis similar to that of coronary ligation [12–14], we inferred that HDAC inhibitors might have cardioprotective effects on reducing infarct size in rats with cardiac IR injury. Additionally, in our laboratory we found that hypoxia-incubated H9c2 cells showed an increase of HDAC6 activities after hypoxic stress (Supplementary Figure 3) (Data not published). H9c2 cells have been demonstrated to be similar to primary cardiomyocytes with regard to energy metabolism patterns and can be successfully used as an in vitro model to simulate cardiac IR injury [15]. Therefore, we inferred that HDAC6 inhibitors might play some roles in cardiac IR injury. Although some studies have also shown positive results of pan-HDAC inhibitors [16, 17], the effects of selective HDAC6 inhibitors on reducing infarct size following cardiac IR injury still remain unclear. Additionally, the evidence of association between HDAC6 inhibition, TGF-β, and HIF-1α is scarce. To address this knowledge gap, in this present study we conducted a computational analysis of high-throughput expression profiling dataset [18] to evaluate the association between HDAC6 inhibitors and gene expression of HIF-1α. The effects of HDAC6 inhibitor ACY1215 on infarct size were determined by using a rat model of cardiac IR injury. Additionally, the association of HDAC6 inhibition and serum expression of TGF-β was also examined.

## Methods

### Analysis of the association between HDAC inhibitors and expression of HIF-1α

In the present study we analyzed the L1000 gene expression dataset in the Connectivity Map [18] which was obtained from the Broad Institute (https://clue.io/data/GCTX#GCTxDatasets). The analysis of dataset generated by treating diverse cell types with pharmacological and genetic perturbagens has been proposed to discover the functional connections between genes, drugs, and diseases [18, 19]. In short, the L1000 is a high-throughput gene expression assay that measures the expression of 978 “landmark” genes from human cells [18] which can be used to computationally infer the expression of 11,350 genes. The data used in the present study was generated in GCTx format, which stored annotated data matrices [20]. The expression level analysis of HIF-1α, the landmark gene in the present study, was obtained from level 5 – moderated Z-score (MODZ). The biological responses of the genome using pan-HDAC inhibitors (i.e., panobinostat and vorinostat) and the HDAC6 inhibitor (i.e., ISOX) were determined for further analysis. In total, we collected 537 data points across 55 different cell lines that were treated with panobinostat, vorinostat, and ISOX.

### HDAC inhibitors and reagents

**Fig. 1:**
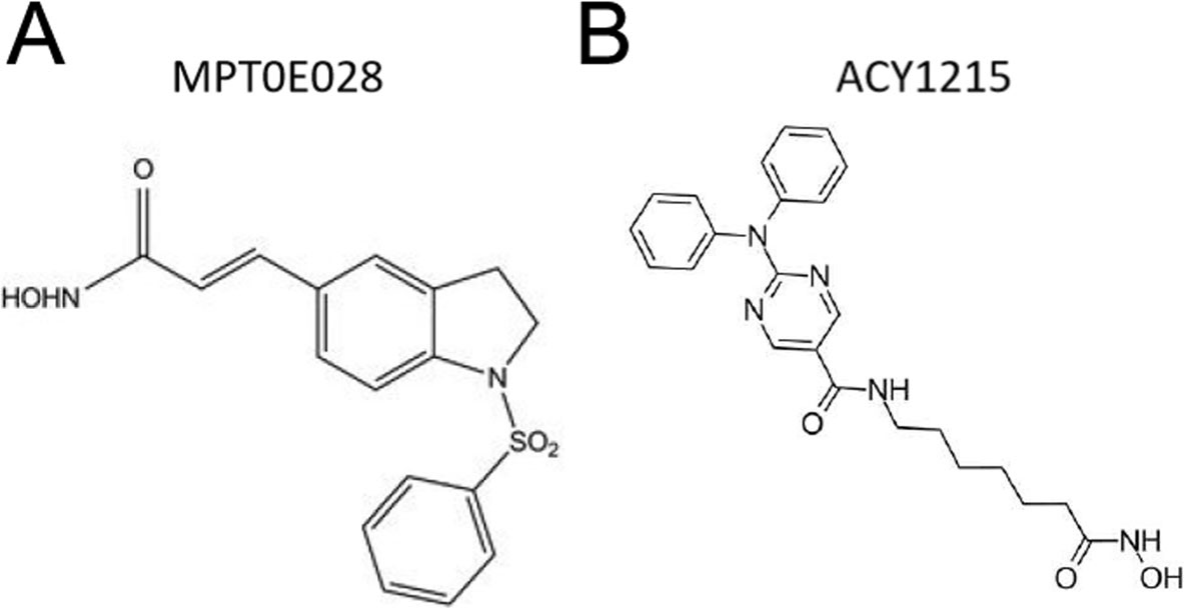
Chemical structures of (**a**) MPT0E028 (3-(1-benzenesulfonyl-2,3-dihydro-1H-indol-5-yl)-N-hydroxy-acrylamide), and (**b**) ACY1215 (N-[7-(hydroxyamino)-7-oxoheptyl]-2-(N-phenylanilino)-5-pyrimidinecarboxamide)

MPT0E028 was a gift from Professor Jing-Ping Liou (School of Pharmacy, College of Pharmacy, Taipei Medical University, Taipei, Taiwan). ACY1215 was purchased from Selleckchem (Houston, TX, USA). The chemical structures of MPT0E028 andACY1215were depicted in Fig. 1a and b, respectively. The 2,3,5-triphenyltetrazolium chloride (TTC) was purchased from Sigma-Aldrich Inc. (St. Louis, MO, USA).

### Cardiac IR injury of rats

All animal experimental procedures were performed under protocols approved by the Institutional Animal Care and Use Committee of Taipei Medical University (IACUC approved No. LAC-2017-0167). Adult male Wistar rats (weighing 200–250 g) were obtained from Laboratory Animal Center of Taipei Medical University and were cared with constant temperature and a 12-h light cycle. The rat model of cardiac IR injury was similar to that of previous studies [16, 21]. Under anesthesia with intraperitoneal injection of pentobarbital sodium (50 mg/kg), animals were intubated and ventilated by using a small animal ventilator (Harvard Apparatus, Holliston, MA, USA). After thoracotomy, the left anterior descending (LAD) coronary artery was ligated by 7–0 silk sutures. Each rat was subjected to ischemia for 45 min followed by reperfusion for 2 h with removal of silk sutures.

### The protocol of HDAC inhibitors administration

**Fig. 2:**
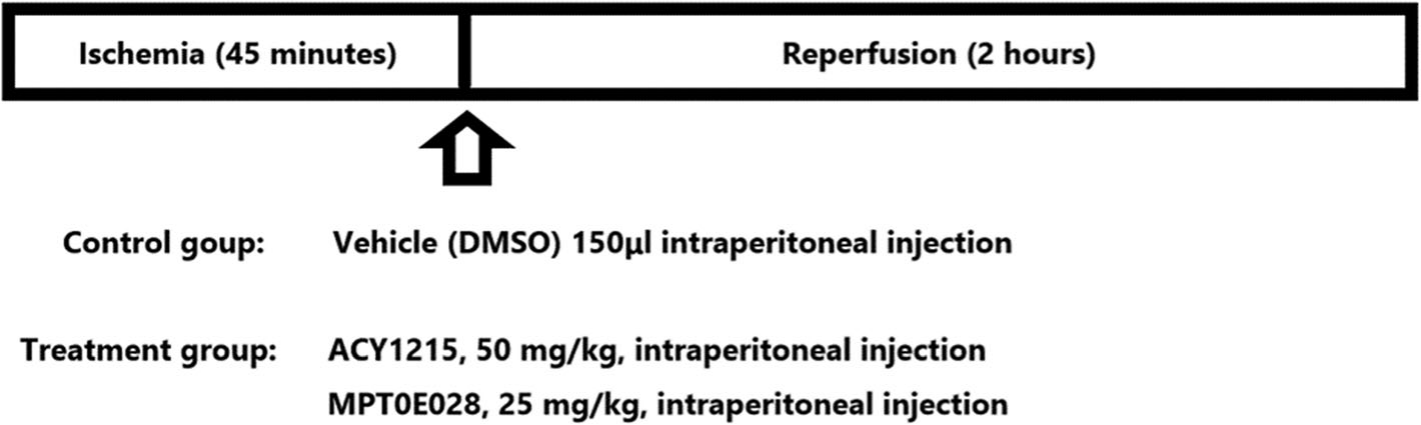
The protocol of cardiac IR injury and administration of test compounds. Adult male Wistar rats (weighing 200–250 g) were intubated and ventilated using a small animal ventilator under anesthesia with intraperitoneal injection of pentobarbital sodium (50 mg/kg). After thoracotomy, the left anterior descending (LAD) coronary artery was ligated by 7–0 silk sutures. Each rat was subjected to ischemia for 45 min followed by reperfusion for 2 h with removal of silk sutures. The dosage of ACY1215 and MPT0E028 used in the present study were 50 mg/kg and 25 mg/kg, respectively. Rats were received treatment by intraperitoneal injection. HDAC inhibitors and vehicle were given within 5 min before reperfusion. After reperfusion for 2 h, rats were euthanized to collect blood and tissue samples for subsequent analysis. DMSO = dimethyl sulfoxide; IR = ischemia-reperfusion; MI = myocardial infarction

The protocol of HDAC inhibitors administration was shown in Fig. 2. Rats were randomized and received vehicle (*n* = 2) or treatment by intraperitoneal injection of ACY1215 and MPT0E028 with the dosage of 50 mg/kg (*n* = 3) and 25 mg/kg (*n* = 3), respectively. HDAC inhibitors and vehicle (dimethyl sulfoxide; DMSO) were given within 5 min before reperfusion. After reperfusion for 2 h, each rat was fixed and venous blood sample was collected via common femoral vein. After blood collection, CO_2_ chamber was used to euthanize the rats and then hearts of rats were excised for subsequent analysis. The process of euthanasia was under regulated of the institutional ethical committee.

### Determination of infarct size

Hearts of rats with cardiac IR injury were excised and perfused by Tyrode’s solution (139 mM NaCl, 3 mM KCl, 17 mM NaHCO_3_, 12 mM glucose, 3 mM CaCl_2_, 1 mM MgCl_2_) to wash out residual blood in the heart chambers. After perfusion with Tyrode’s solution, the previous occluded LAD was ligated again and the heart was perfused with 1% methylene blue in which the area without blue staining was defined as “area at risk” (AAR). After methylene blue staining, the heart was treated by TTC staining for determination of infarct size. TTC is a white compound and enzymatically reduced to red 1,3,5-triphenylformazan (TPF) in living tissues by dehydrogenases, while it remains as white TTC in necrotic areas. After TTC staining, the white area was defined as infarction area (IA). In the present study, the infarct size was presented as the ratio of IA to AAR (IA/AAR).

### Enzyme-linked immunosorbent assays (ELISA)

Blood samples of rats with cardiac IR injury were collected to determine the serum expression of TGF-β and C-reactive protein (CRP) by ELISA (Signosis Inc., Santa Clara, CA, USA). Briefly, whole blood from rats with cardiac IR injury was collected and then centrifuged at 3000 rpm for 10 min at 4 °C. The aliquot supernatant (serum) was added 100 μl into the well incubating overnight at 4 °C. After the reaction, wells were washed with wash buffer and then added 100 μl of detection antibody incubating for 1 h at 37 °C. Then wells were washed again and then added 100 μl of anti-rat IgG HRP-linked antibody incubating for 30 min at 37 °C. After incubation, we washed the wells once again and then added 100 μl TMB (3, 3′, 5, 5″-tetramethylbenzidine) substrate for color production, and finally added 100 μl STOP solution to terminate the reaction. The results were measured by spectrophotometer at wavelength 450 nm.

### Statistics

The serum expression of TGF-β and CRP in rats treated with HDAC inhibitors was compared to that of rats treated with vehicle. After TTC staining, the infarct size and the ratio of AAR/V in excised hearts of rats with cardiac IR injury were compared between groups. Categorical data were presented as mean ± S.E.M. or as percentage of basal. Statistical comparisons between groups were performed using the student’s t-test or one-way ANOVA. *P* < 0.05 was considered statistically significant.

## Results

### HDAC inhibitors were associated with a decrease in expression of HIF-1α

**Fig. 3:**
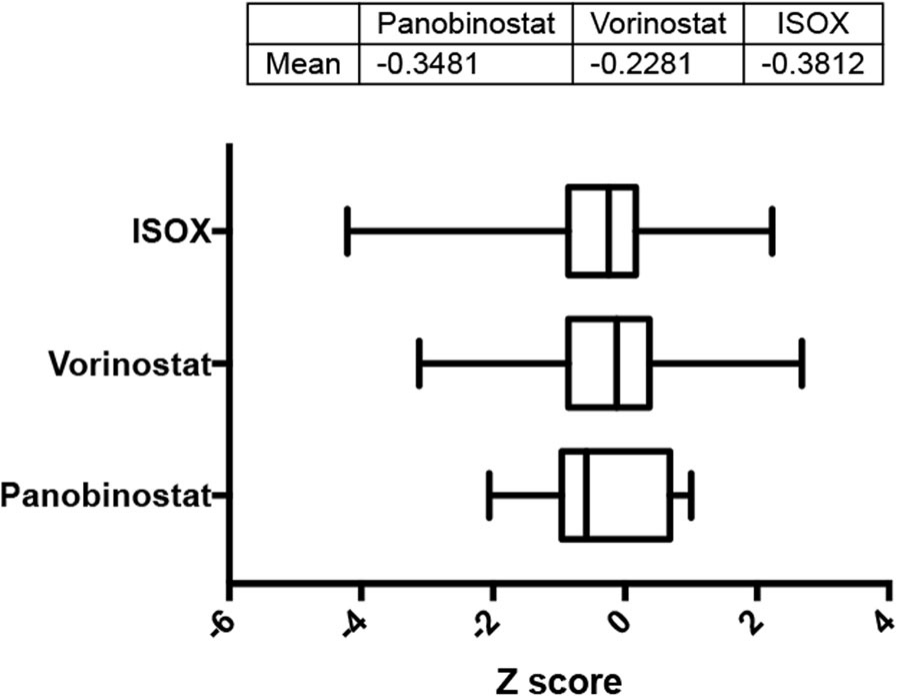
The association between HDAC inhibitors and HIF-1α expression by analyzing L1000 gene expression dataset in the Connectivity Map

Our analysis found that the expression level of HIF-1α was decreased upon treatment with the three HDAC inhibitors in a time duration of 6 h (Fig. 3). Of these inhibitors, ISOX showed the greatest decrease in HIF-1α expression (Fig. 3). This analysis indicated that HDAC inhibitors might play roles in cardiac IR injury. Based on these mentioned findings, we conducted further in vivo experiments to investigate the effects of ACY1215 and MPT0E028 on infarct size in rats with cardiac IR injury.

### Administration of ACY1215 and MPT0E028 attenuated infarct size in rats with cardiac IR injury

**Fig. 4:**
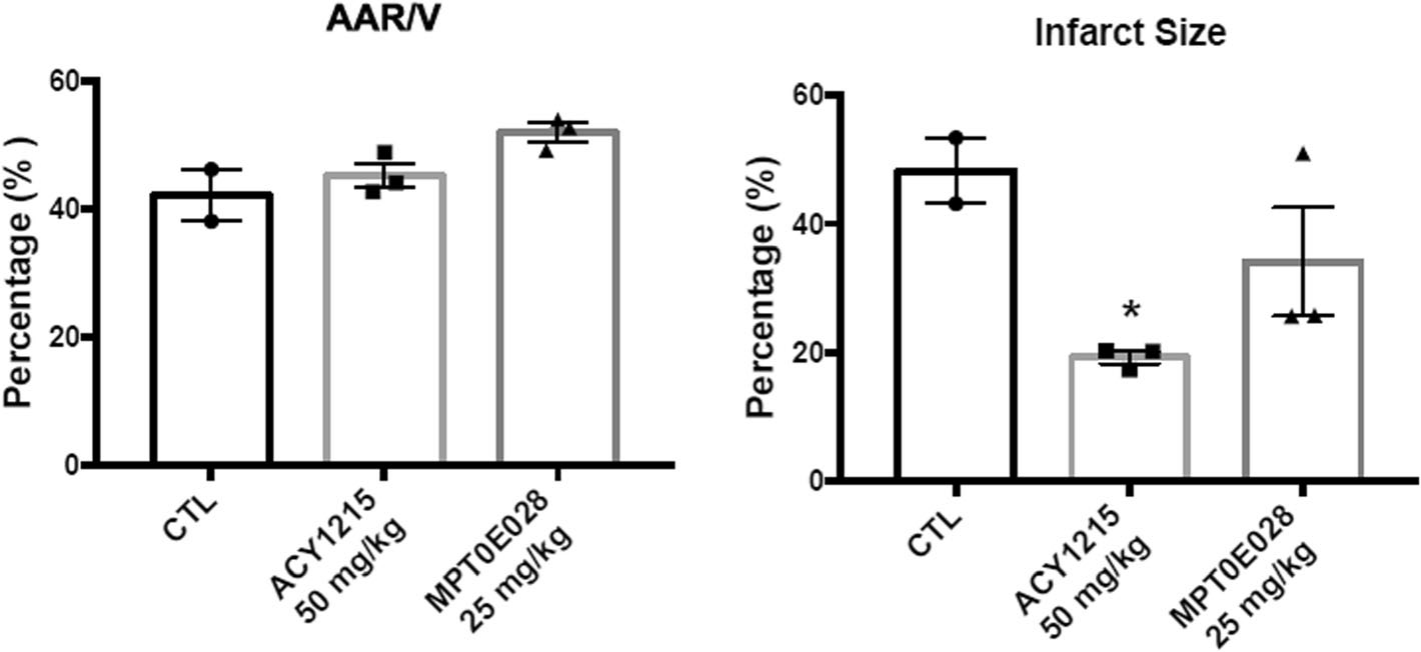
The ratios of AAR/V and infarct size in rats of control and treatment groups. AAR = area at risk; CTL = control; V = ventricular weight

The ratios of AAR/V were comparable without statistical significances between groups (42.09 ± 5.71%, 45.15 ± 3.23%, and 51.91 ± 2.47% in control, ACY1215, and MPT0E028 groups, respectively) (Fig. 4). In addition, we observed that rats treated with ACY1215 had a significant smaller infarct size in the myocardium compared to control rats (19.22 ± 1.70% vs. 49.75 ± 9.36%, *p* < 0.05). Although administration of MPT0E028 in rats led to a trend of reducing infarct size (34.16 ± 14.58%) compared to that in control rats, the trend did not reach a statistical significance (Fig. 4).

Based on the findings of the effects of HDAC inhibitors on infarct size, we conducted further survey to determine the association between HDAC inhibitors and the serum expression of MI-related biomarkers, TGF-β and CRP.

### Administration of ACY1215 and MPT0E028 reduced the expression of serum TGF-β in rats with cardiac IR injury

**Fig. 5:**
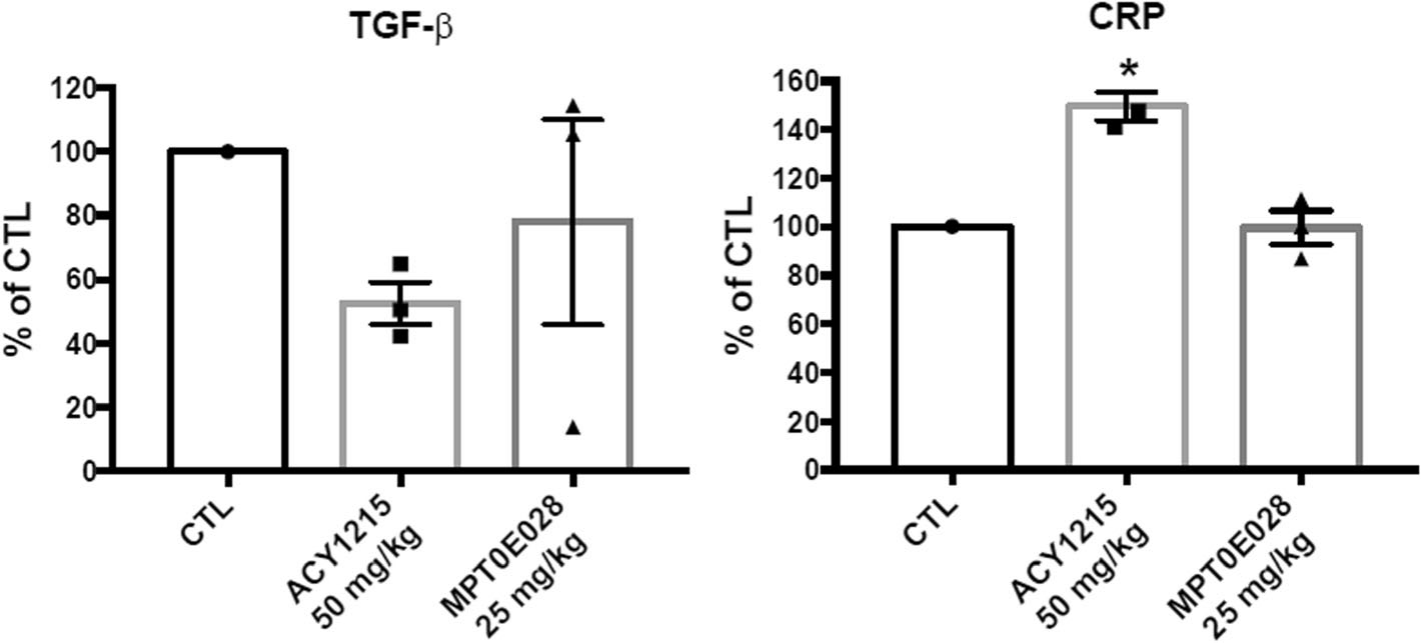
The expression of serum TGF-β and CRP in IR rats treated with HDAC inhibitors compared to control rats. CRP = C-reactive protein; CTL = control; TGF-β = transforming growth factor-β

Although there was a trend of decreased serum TGF-β levels of rats treated with ACY1215 and MPT0E028 compared to that of control rats, it did not reach a statistical significance (8960 pg/mL, 4713.33 ± 1027.34 pg/mL, and 6990 ± 4987.05 pg/mL in control, ACY1215, and MPT0E028 groups, respectively) (Fig. 5).

### Administration of ACY1215 enhanced the expression of serum CRP in rats with cardiac IR injury

The onset of acute ischemia in the setting of MI induces aggressive inflammatory responses [8]. To evaluate the effects of HDAC inhibitors on inflammation, the serum expression of CRP was also determined. Compared to control rats, rats treated with ACY1215 had a significant enhanced expression of serum CRP (641.6 μg/mL vs. 961.37 ± 64.94 μg/mL, *p* < 0.05). In contrast to rats treated with ACY1215, rats treated with MPT0E028 did not have a significant change in the expression of serum CRP compared to control rats (641.6 μg/mL vs. 638.32 ± 78.48) (Fig. 5).

## Discussion

The main findings in the present study implicated that ACY1215 administered before reperfusion might be a potential pharmacological strategy to attenuate infarct size during cardiac IR injury. The possible mechanism of ACY1215-mediated cardioprotective effects might be related to modulating HIF-1α expression. Additionally, the use of ACY1215 to limit infarct size should be under cautious monitoring to avoid eliciting additional inflammation.

In one study which was conducted by using an ex vivo IR model, the selective HDAC6 inhibitor tubastatin was reported to result in no benefit to limit infarct size [22]. However, in another study tubastatin administered for 7 days before ligation of coronary arteries showed a cardioprotective effect to reduce infarct size in rats with cardiac IR injury [23]. These inconsistent results of previous studies might be due to different protocols of drug administration and experimental IR models used in each study. The findings of the present study showed that inhibition of HDAC6 activities in the interval between ischemia and reperfusion effectively limited infarct size in rats with cardiac IR injury. The mechanisms in which ACY1215 reduced infarct size still remained unclear. It has been proposed that inhibition of HDAC6 activities led to a reduced infarct size by attenuation of reactive oxygen species and upregulation of peroxiredoxin 1 acetylation [23]. Additionally, ACY1215 might reduce infarct size by inhibiting the expression of HIF-1α based on the results of the present study. Some previous studies [5, 6] provided supportive evidence to our inference. However, some other studies [24–26] showed opposite results that activation of HIF-1α expression conferred a cardioprotective effect to diminish cardiac IR injury. Despite these mentioned inconsistent results, our findings in the present study implicated the possible effects of HDAC6 inhibitors on HIF-1α expression and cardiac IR injury. Further studies are necessary to clarify the exact roles of HIF-1α involved in ACY1215-mediated reduction of infarct size following cardiac IR injury.

The cytokine TGF-β has been shown to involve in the process of cardiac remodeling [27] after ischemic insults, including cardiomyocytes apoptosis [28] and proliferation of cardiac fibroblasts [29]. Additionally, inhibition of HDAC6 activities has been reported to suppress TGF-β-mediated proliferation of cardiac fibroblasts and post-MI cardiac fibrosis [30]. However, the benefits of inhibiting TGF-β signaling after MI remain controversial [27, 31]. Inhibition of TGF-β in the late post-MI period attenuated cardiac remodeling and interstitial fibrosis, while inhibition of TGF-β in the early post-MI period resulted in disruption of myocardial healing [4] and an increased risk of cardiac rupture [32]. The findings in the present study showed that the use of ACY1215 not only reduced infarct size but also avoided posing an additional risk of cardiac rupture. Collectively, these mentioned findings suggest that pharmacological interventions targeting TGF-β in cardiac IR injury should be implemented cautiously. Further investigation is needed to determine the adequate protocol of HDAC inhibitors administration, including dosage and timing, that provide a cardioprotective effect in reducing infarct size and subsequent cardiac fibrosis without an increased risk of adverse cardiac events following MI.

The role of CRP involved in the disease course of MI has been widely studied and still debated. Some therapeutic interventions to inhibit CRP-related signaling in experimental IR models did not show a limited infarct size [33, 34]. Additionally, clinical studies which focused on the effects of suppressing serum CRP levels on preventing adverse cardiac remodeling showed inconsistent results [35, 36]. The results in the present study indicated that a reduction in infarct size associated with ACY1215 might be not processed by inhibition of CRP-related signaling. Additionally, our findings also implicated that the use of ACY1215 should be administered cautiously to avoid an additional inflammation. Collectively, we suggested that serum levels of TGF-β and CRP might be candidate biomarkers to monitor the safety and efficacy of HDAC inhibitors administered in cardiac IR injury.

In contrast to ACY1215, administration of MPT0E028 did not significantly limit infarct size in rats with cardiac IR injury. The potential reasons might be the relatively small sample size used in the present study. Despite a neutral effect was observed, MPT0E028 showed insignificant influences on serum expression of TGF-β and CRP. These mentioned results showed that MPT0E028 might be still a potential candidate for further survey focusing on reducing infarct size without increased risks of cardiac rupture or additional inflammation following MI.

To the best of our knowledge, the present study was the first report to demonstrate the cardioprotective effects of selective HDAC6 inhibitor ACY1215 on diminishing infarct size in experimental IR models. However, our study is subjected to some limitations. First, we acknowledged that the very small sample size was a serious limitation of our study. Therefore, the data in the present study should be interpreted carefully and cautiously. Second, we did not examine the effects of HDAC inhibitors on immune cells that were also involved in the pathogenesis of cardiac IR injury. In addition, we did not investigate and compare the effects of HDAC inhibitors in different dosage and timing of administration. Third, we did not examine other well-known biomarkers following MI (e.g., creatine kinase and NT-proBNP) and hemodynamic parameters in the animal experiments for comparison. Fourth, the rat IR model used in the present study did not fully reflect the actual clinical situations. Additionally, changes in ambient oxygen concentrations and variations of oxygen diffusion in tissues have a strong impact on HIF-1α stabilization when working in vivo because of the ultrashort half-life (< 5 min) of HIF-1α in oxygenated atmosphere [37]. These unstable natures of HIF-1α became big obstacles in the present study to examine the expression of HIF-1α in blood samples and heart tissues of rats. Despite these limitations, the results presented in this study propose a promising implication for future investigation in the field of drug discovery and provide valuable data that shows the potential pharmacological role of ACY1215 in reducing infarct size following cardiac IR injury. Additionally, our findings highlight that the safety and efficacy of HDAC inhibitors should be evaluated carefully by detection of serum TGF-β and CRP levels.

## Conclusions

Taken these data together, the selective HDAC6 inhibitor ACY1215 showed a beneficial effect on reducing infarct size in rats with cardiac IR injury by a possible mechanism of inhibition in HIF-1α expression. TGF-β and CRP should be useful biomarkers to monitor the use of ACY1215 in cardiac IR injury. These findings might be of clinical importance in applying ACY1215 as a novel pharmacological intervention to attenuate cardiac IR injury and preserve ventricular function following MI.

## Supplementary information

**Additional file 1: Supplementary Figure 1.** Protocol of MPT0E028 administration in ISO-treated rats. BID = twice per day; CTL = control; DMSO = dimethyl sulfoxide; IP = intraperitoneal; ISO = isoproterenol; PO = per os (by mouth); QD = once per day. **Supplementary Figure 2.** The serum levels of NT-proBNP and collagen area in myocardium of ISO-treated rats. MPT0E028 administration significantly reduced the serum NT-proBNP levels and collagen area in myocardium in ISO-treated rats. BID = twice per day; CTL = control; ISO = isoproterenol; NT-proBNP=N-terminal prohormone of brain natriuretic peptide; QD = once per day. ^*^p < 0.05, ^**^p < 0.01, ^***^p < 0.001, ^****^p < 0.0001. **Supplementary Figure 3.** HDAC6 activities in hypoxia-incubated H9c2 cell. Hypoxia-incubated H9c2 cells showed a decreased expression of acetyl-α-tubulin (AC-tubulin) compared to normoxia-incubated H9c2 cells, indicating the increase of HDAC6 activities after hypoxic stress. AC-tubulin = acetyl-α-tubulin; H = hour; H = hypoxia; HDAC = histone deacetylase; min = minutes; N = normoxia.

## Data Availability

The dataset supporting the conclusions of this article, including all figures in the text, is included within the article and submitted. The protocol for this study was approved by the Institutional Animal Care and Use Committee of Taipei Medical University. Thus, access to the data will be subject to approval by the Institutional Review Board of Taipei Medical University.

## Abbreviation

AAR: Area at risk
CRP: C-reactive protein
ELISA: Enzyme-linked immunosorbent assays
HDAC: Histone deacetylase
HIF-1α: Hypoxia inducible factor-1α
IA: Infarction area
IR: Ischemia-reperfusion
LAD: Left anterior descending
MI: Myocardial infarction
MODZ: Moderated Z-score
TGF-β: Transforming growth factor-β
TMB: 3, 3′, 5, 5″-tetramethylbenzidine
TPF: 1,3,5-triphenylformazan
TTC: 2,3,5-triphenyltetrazolium chloride
