# Supplementary material for: Investigating the potential effects of selective histone deacetylase 6 inhibitor ACY1215 on infarct size in rats with cardiac ischemia-reperfusion injury
Source: BMC Pharmacol Toxicol. 2020 Mar 12;21:21. doi: 10.1186/s40360-020-0400-0 (PMC7077123; doi:10.1186/s40360-020-0400-0)
Supplement: Supplementary file 1 — Additional file 1: Supplementary Figure 1. Protocol of MPT0E028 administration in ISO-treated rats. BID = twice per day; CTL = control; DMSO = dimethyl sulfoxide; IP = intraperitoneal; ISO = isoproterenol; PO = per os (by mouth); QD = once per day. Supplementary Figure 2. The serum levels of NT-proBNP and collagen area in myocardium of ISO-treated rats. MPT0E028 administration significantly reduced the serum NT-proBNP levels and collagen area in myocardium in ISO-treated rats. BID = twice per day; CTL = control; ISO = isoproterenol; NT-proBNP=N-terminal prohormone of brain natriuretic peptide; QD = once per day. *p < 0.05, **p < 0.01, ***p < 0.001, ****p < 0.0001. Supplementary Figure 3. HDAC6 activities in hypoxia-incubated H9c2 cell. Hypoxia-incubated H9c2 cells showed a decreased expression of acetyl-α-tubulin (AC-tubulin) compared to normoxia-incubated H9c2 cells, indicating the increase of HDAC6 activities after hypoxic stress. AC-tubulin = acetyl-α-tubulin; H = hour; H = hypoxia; HDAC = histone deacetylase; min = minutes; N = normoxia. [file 40360_2020_400_MOESM1_ESM.docx]

**Supplementary Materials**


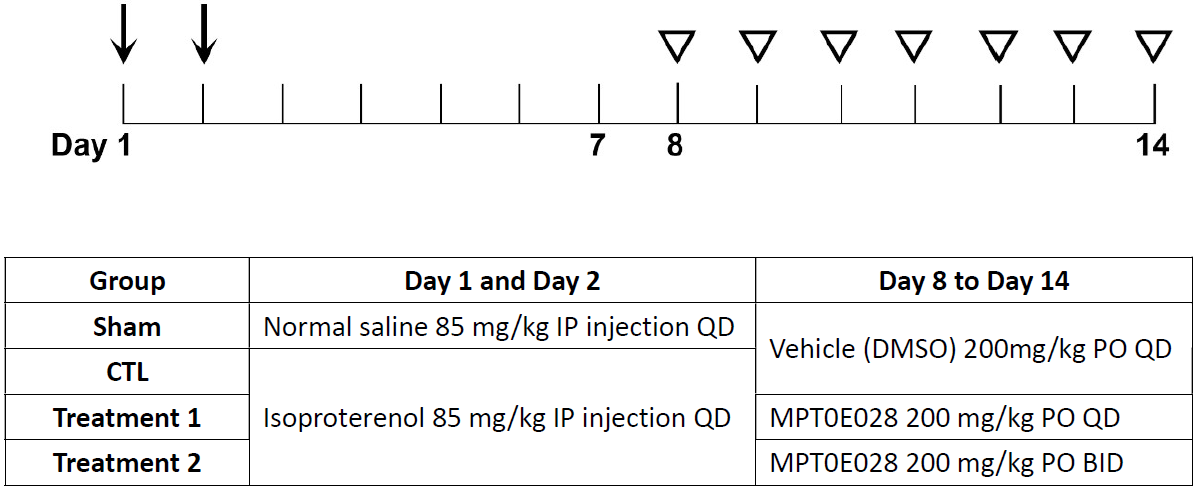


Supplementary Figure 1. Protocol of MPT0E028 administration in ISO-treated rats. BID=twice per day; CTL=control; DMSO=dimethyl sulfoxide; IP=intraperitoneal; ISO=isoproterenol; PO=per os (by mouth); QD=once per day.


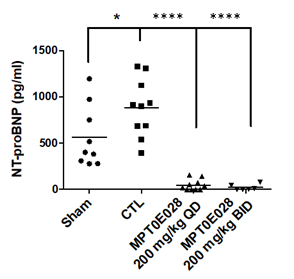

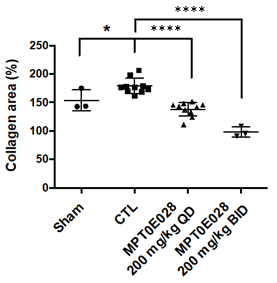


Supplementary Figure 2. The serum levels of NT-proBNP and collagen area in myocardium of ISO-treated rats. MPT0E028 administration significantly reduced the serum NT-proBNP levels and collagen area in myocardium in ISO-treated rats. BID=twice per day; CTL=control; ISO=isoproterenol; NT-proBNP=N-terminal prohormone of brain natriuretic peptide; QD=once per day. ^*^p<0.05, ^**^p<0.01, ^***^p<0.001, ^****^p<0.0001.


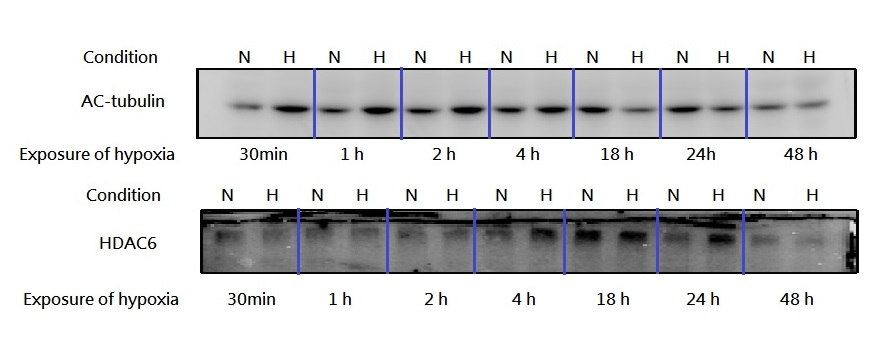


Supplementary Figure 3. HDAC6 activities in hypoxia-incubated H9c2 cell. Hypoxia-incubated H9c2 cells showed a decreased expression of acetyl-α-tubulin (AC-tubulin) compared to normoxia-incubated H9c2 cells, indicating the increase of HDAC6 activities after hypoxic stress. AC-tubulin= acetyl-α-tubulin; H=hour; H=hypoxia; HDAC=histone deacetylase; min=minutes; N=normoxia.
